# Psychosocial predictors of consistent condom use among migrant road construction workers in the Southwest Region of Cameroon using the Health Belief Model

**DOI:** 10.11604/pamj.2018.29.215.15130

**Published:** 2018-04-18

**Authors:** Elvis Enowbeyang Tarkang, Lilian Belole Pencille

**Affiliations:** 1 School of Public Health, University of Health and Allied Sciences, Ho, Volta Region, Ghana; 2 HIV/AIDS Prevention Research Network Cameroon (HIVPREC), Kumba, South-west Region, Cameroon

**Keywords:** Consistent condom use, Health Belief Model (HBM), HIV/AIDS, migrant road construction workers, Cameroon

## Abstract

**Introduction:**

A significant proportion of road construction workers are migrants and prone to HIV infection. This study investigated the psychosocial predictors of consistent condom use among migrant road construction workers in the South-west region of Cameroon using the Health Belief Model (HBM) as the theoretical framework.

**Methods:**

A cross-sectional survey of a stratified sample of 254 road construction workers was conducted at construction sites along the Kumba-Mamfe road in the South-west region of Cameroon in December 2015. Data were collected using a pretested structured questionnaire and binomial logistic regression was performed to test the strength of association between the independent and the dependent variables using SPSS version 20 at the level 0.05.

**Results:**

Only 67 (43.5%) reported consistent condom use. Perception of risk of contracting HIV was also low, 109 (42.9%). None of the constructs of the HBM was significantly associated with consistent condom use. However, perception that road construction workers are prone to HIV (perceived susceptibility) was associated with an increased likelihood of using condom consistently, OR = 2.1 (95% CI 0.72-6.12, p = 0.17); perception that consistent condom use could prevent HIV transmission (perceived benefit) was associated with an increased likelihood of using it consistently, OR = 1.9 (95% CI 0.74-4.80, p = 0.18); perception by workers that they can refuse sex with their partners if they refuse to use condoms (perceived self-efficacy) was associated with an increased likelihood of using condoms consistently, OR = 1.5 (95% CI 0.62-3.53, p = 0.38). However, the perception that condom reduces sexual pleasure (perceived barriers) was associated with a reduced likelihood of using it consistently, OR = 0.84 (95% CI 0.35-2.01, p = 0.698)

**Conclusion:**

There were no significant associations between the psycho-social constructs of the HBM and consistent condom use. Therefore, interventions to increase the perception of risk of contracting HIV, which is assumed to be the immediate antecedent of consistent condom use is highly recommended.

## Introduction

There is enough global evidence on the vulnerability of construction workers to HIV/AIDS. An International Labour Organization (ILO) Report in 2007 listed a number of work and lifestyle factors which expose construction workers to the risk of HIV infection, depending upon their working situations: high mobility, isolation and working in confined environments with limited contacts, very young adults or in sexually active age group, access to and ready availability of sex workers and inadequate access to health services. A significant proportion of these workers are migrants and prone to HIV infection [1]. These migrant road construction workers are most likely to carry the infection and pass on to others at their source communities and others along their migratory route. Sub-Saharan Africa (SSA) is the region hardest hit by HIV/AIDS and the main risk factors for HIV transmission are migration, poverty, unequal distribution of wealth, lack of education, inequalities and various cultural influences [2, 3]. In most SSA countries, migration and HIV/AIDS are linked to heterosexual transmission, fuelled by rampant sexually transmitted infections (STI), multiple and commercial sexual relationships, inconsistent condom use, poor access to health service and other social-cultural and economic factors related to migration [4, 5]. Large construction sites offer job and business opportunities that attract a large number of young people, many of whom migrate from poor and rural areas of nearby regions. Construction workers comprise one of the key mobile groups together with truckers, transport workers, and itinerant traders. HIV/AIDS continues to ravage families and communities in SSA with those between the ages of 19 and 55 years being the hardest hit group. This age group coincides with the most productive group in Africa, especially workers of road construction sites. Norms and practices pertaining to social and sexual life among workers aged 19-55 years is that of ambivalence about sexual experimentation, unprotected casual sex, multiple sexual partners and similar high-risk activities such as drug and substance abuse. These activities are not only dangerous but increase the risk of the workers to STIs including HIV/AIDS.

Environmental influences like urban lifestyle, low socioeconomic status, peer pressure and other social vices are some of the factors that facilitate infection among road construction workers and the populace. Despite the recently reported decrease in the HIV/AIDS prevalence to 4.3%, Cameroon remains on the list of countries with the highest overall HIV prevalence in West and Central Africa. Prevalence remains relatively high among long-distance truck drivers, female commercial sex workers and other most-at-risk populations [6]. The main mode of HIV transmission in Cameroon is sexual intercourse, accounting for 90% of new infections [7]. However, prevention is the first line of defense against HIV/AIDS and the correct and consistent use of condom is a mainstay of HIV prevention approaches [8]. Migration, mobility and HIV/AIDS are well-documented interlinked phenomena. High rates of HIV infections are generally found along transport routes and in regions experiencing high seasonal and long-term population mobility as that found in the Kumba-Mamfe road construction sites in the South-west region of Cameroon. The location of the municipalities concerned (Kumba III, Konye, Nguti, Mamfe and Tinto) in the South-west Region, the third highest HIV-infected region of Cameroon, demands serious attention. The Health Belief Model (HBM) theorizes that people's beliefs about whether or not they are at risk for a disease or health problem (HIV/AIDS) and their perceptions of the benefits of taking action to avoid it (consistent condom use), influence their readiness to take action (use condoms consistently during sex to prevent HIV/AIDS) [9, 10]. The HBM asserts that the motivation for people to take action to promote or prevent a disease is based on how strongly they believe that they are susceptible to the disease in question; whether the disease would have serious effects on their lives if they should contract it; whether the suggested health intervention is of value; whether the effectiveness of the treatment is worth the cost; which barriers people must overcome to institute and maintain specific behaviours; influence by another person close by, who may have been susceptible to the same disease, signaling the need for action. No study has been conducted in Cameroon on condom use among road construction workers. This paper uses the main constructs of the HBM, as the theoretical framework to investigate the psychosocial predictors of consistent condom use among migrant road construction workers in road construction sites along Kumba-Mamfe road in the South-west region of Cameroon, in order to inform an HIV/AIDS prevention program among them. In this study, it is hypothesized that migrant road construction workers would use condoms consistently during sexual intercourse to prevent HIV if they perceive themselves to be at risk of contracting HIV/AIDS.

## Methods

**Research design**: A cross-sectional survey was conducted among 254 migrant road construction workers at construction sites along the Kumba-Mamfe road in the South-west region of Cameroon in December 2015. Data were collected using a pretested structured questionnaire.

**Study setting**: The study was conducted at the two construction sites along the Kumba-Mamfe road in the South-west region of Cameroon. This road passes through five municipalities in the South-west region of Cameroon: Kumba III, Konye, Nguti, Tinto and Mamfe municipalities, covering a considerable expanse of land. The South-west region has an HIV/AIDS prevalence of 5.7%, which is above the Cameroon national prevalence of 4.3% [11].

**Study population**: The population for this study comprised male construction workers at the two road construction sites along the Kumba-Mamfe road in the South-west region of Cameroon made up of Drivers, Builders, Welders, Iron Benders and unskilled Laborers.

**Sampling method**: A proportionate stratified sampling approach was used whereby for each construction site, a list of all employees was obtained along with their types of work. The research team then attempted to sample a proportionate number of employees from each site in order to make up the predetermined sample size for each construction site. Workers of the HIV/AIDS Prevention Research Network, Cameroon (HIVPREC), acting as peer educators explained to the workers what the survey was all about. Those who were interested consented to participate.

**Data collection**: A pre-tested structured questionnaire was used to collect data for this study. The questionnaire was pretested on a convenience sample of 10 migrant road construction workers who did not take part in the actual study. The questionnaires were administered by trained facilitators of HIVPREC, due to the perceived low level of literacy of the respondents. The validity of the questioned was ensured by designing items to measure the different variables of the study by the first author who is a specialist in HIV research. The questionnaire captured data on socio-demographic characteristics (age, marital status, religion, educational level and profession), constructs of the HBM (perceived susceptibility to HIV, perceived severity of HIV, perceived benefit of condom use, perceived barriers to condom use, perceived self-efficacy for condom use and perception of risk of contracting HIV) and condom use. Previous studies guided the development of the questionnaires [12, 13]. Completed questionnaires were checked by the HIVPREC facilitators, to ensure the completeness and consistency of the data.

**Ethical considerations**: Respondents were given verbal and written information about the study and signed an informed consent form before participation in the study. No personal or identifying information was retained in the questionnaire. All respondents participated on a voluntary basis and no financial incentives were provided. Permission to conduct this study was obtained from research and ethics committee of the HIV/AIDS Prevention Research Network, Cameroon (HIVPREC) and from the Management of the Kumba-Mamfe road project in the South-west region of Cameroon. Ethical clearance for this study was obtained from the research and ethics committee of the faculty of philosophy, religious and social studies of the Cameroon Christian University (CCU).

### Measures

#### Outcome (dependent) variable: consistent use of male condom

**The regularity of condom use**: This was measured with the following question: "How often do you use a condom during sexual intercourse" and it was asked only to the sexually active workers. The response options were "1 = others" (reference category)' and "2 = always".

#### Predictor (independent) variables: constructs of the HBM

**Perceived susceptibility to HIV**: This was measured based on the level of agreement with the following item: "Road construction workers are prone to HIV/AIDS". The response options were categorized into "2 = agree" and "1 = disagree". "Disagree" was coded as the index category.

**Perceived severity of HIV/AIDS**: This measure was based on the degree of agreement with the following statement: "HIV/AIDS is a deadly disease". The response options were categorized into "2=agree" and "1 = disagree". "Disagree" was coded as the index category.

**Perceived benefit of condom use**: This was measured based on the level of agreement with the following item: "Consistent condom use during sexual intercourse can prevent HIV transmission". The response options were categorized into "2 = agree" and "1 = disagree". "Disagree" was coded as the index category.

**Perceived barriers to condom use**: This measure was based on the degree of agreement with the following statement: "Condom use decreases sexual pleasure". The response options were categorized into "2 = agree" and "1=disagree". "Disagree" was coded as the index category.

**Perceived self-efficacy for condom use**: This measure was based on the degree of agreement with the following statement: "I feel confident to refuse sex with my partner(s) if they refuse to use condom". The response options were categorized into "2 = agree" and "1 = disagree". "Disagree" was coded as the index category.

**Socio-demographic variables**: The following socio-demographic variables were included in the study: age, categorized into five groups (10-19, 20-29, 30-39, 40-49 and 50 and above years), marital status, categorized into three groups (single, married and others), Religion, categorized into three groups (Christian, Muslim and others), Educational level categorized into four groups (No education, primary, secondary/high school and tertiary) and occupation, categorized into Driving, Building, Iron bending, Welding and Unskilled labour.

**Perception of risk of contracting HIV**: This was measured with the following question: "How at risk of contracting HIV are you?" The response options were "1=not at risk (reference category)" and "2=at risk".

**Data analysis**: Frequencies, percentages and binomial logistic regression were performed using SPSS version 20 software program at the level 0.05, to examine the likelihood of using the condom consistently during sexual intercourse. Binomial logistic regression predicts the probability that an observation falls into one of two categories of a dichotomous dependent variable based on one or more independent variables that can be either continuous or categorical. The procedure gives rise to estimates of odds of a certain event occurring (consistent condom use), given a set of explanatory variables (psychosocial and demographic factors). To estimate the odds ratios (OR), we built different models predicting consistent condom use during sexual intercourse, using the various constructs of the HBM and the socio-demographic variables. To assess the predictive utility of each construct of the HBM as a whole model, that is how individuals with various combinations of health beliefs are more or less likely to consistently use the condom during sexual intercourse, each component of the HBM was entered into the model one at a time. The significant level for all statistical tests was 5%.

## Results

**Socio-demographic characteristics**: The majority of the respondents, 219 (86.2%) were aged 20-49 years; most, 212 (83.5%) were Christians; most, 186 (73.2%) were single and the majority, 170 (66.9%) had up to high school level of education (Table 1).

**Table 1:** Socio-demographic characteristics of the study population

Characteristics	Frequency	Percentage
Age Group		
10-19	19	7.5
20-29	106	41.7
30-39	74	29.1
40-49	39	15.4
50 and above	16	6.3
Religious Affiliation		
Christian	212	83.5
Muslim	27	10.6
Others	15	5.9
Marital Status		
Single	186	73.2
Married	47	18.5
Others	21	8.3
Educational Attainment		
No education	81	31.9
Primary	92	36.2
Secondary/High school	78	30.7
Tertiary	3	1.2
Occupation		
Driving	16	6.3
Building	70	27.6
Iron bending	55	21.6
Welding	43	16.9
Unskilled labour	70	27.6

**Psychosocial predictors of consistent condom use**: The psychosocial predictors of consistent condom use in this study were the constructs of the HBM (Table 2). The majority of the respondents, 203 (79.9%) perceived that road construction workers are prone to HIV (perceived susceptibility to HIV). However, the proportion of workers who perceived that HIV is deadly (perceived severity of HIV/AIDS) and that consistent use of condom during sexual intercourse can prevent HIV transmission (perceived benefit of condom use), were low, 163 (64.2%) and 159 (62.6%) respectively. The majority, 137 (63.8%) perceived that condom use decreases sexual pleasure (perceived barrier to condom use) and few 137 (53.9%) perceived that they feel confident to refuse sex with their partners if they refuse to use condom (perceived self-efficacy for condom use). Only few, 109 (42.9%) perceived themselves to be at risk of contracting HIV, and in the same vein, only few of the sexually active workers, 67 (43.5%) used condom consistently during sexual intercourse. None of the constructs of the HBM and the socio-demographic variables was a significant predictor of consistent condom use. However, perception that road construction workers are prone to HIV was associated with an increased likelihood of using condom consistently, (OR = 2.1 (95% CI 0.72-6.12, p=0.17)); perception that consistent condom use could prevent HIV transmission was associated with an increased likelihood of using it consistently, (OR = 1.9 (95% CI 0.74-4.80, p = 0.18)); perception by workers that they can refuse sex with their partners if they refuse to use condom was associated with an increased likelihood of using condom consistently, (OR = 1.5 (95% CI 0.62-3.53, p = 0.38)); perception that condom reduces sexual pleasure was associated with a reduced likelihood of using it consistently, (OR = 0.84 (95% CI 0.35-2.01, p=0.698)); perception of being at risk of contracting HIV was associated with an increased likelihood of using condom consistently, (OR=1.30 (95% CI 0.55-2.95, p = 0.5770)); being a Muslim was associated with an increased likelihood of using condom consistently, (OR = 1.60 (95% CI 0.20-12.54, p = 0.66)) and being single was associated with an increased likelihood of using condom consistently, (OR=2.50 (95% CI 0.38-17.06, p=0.33)) (Table 3).

**Table 2:** Psychosocial predictors of consistent condom use

Psychosocial predictors	Frequency	Percentage
**Road construction workers are prone to HIV**		
Agree	203	79.9
Disagree	51	20.1
**HIV/AIDS is a deadly disease**		
Agree	163	64.2
Disagree	91	35.8
**Consistent condom use can prevent HIV transmission**		
Agree	159	62.6
Disagree	95	37.4
**Condom use decreases sexual pleasure**		
Agree	162	63.8
Disagree	92	36.2
**I feel confident to refuse sex with my partner(s) if they refuse to use condom**		
Agree	137	53.9
Disagree	117	46.1
**Perception of risk of contracting HIV**		
At risk	109	42.9
Not at risk	145	57.1
**Regularity of condom use**		
Always	67	43.5
Others	87	56.5

**Table 3:** Odds Ratios (OR) of using condom consistently from the logistic regression models

Effect	Odds Ratios (OR)	Confidence interval (CI)	Wald	Sig.
**Perceived Susceptibility**				
Road construction workers are prone to HIV/AIDS	2.1	(0.722-6.115)	1.854	0.173
**Perceived severity**				
HIV/AIDS is a deadly disease	0.595	(0.252-1.408)	1.390	0.238
**Perceived benefit of condom use**				
Consistent use of condom can prevent the sexual transmission of HIV	1.90	(0.741-4.797)	1.769	0.183
**Perceived barriers to condom use**				
Condoms reduce sexual pleasure	0.84	(0.352-2.012)	0.151	0.698
**Perceived condom use self-efficacy**				
I feel confident that I can refuse my partner(s) if they refuse to use condoms during sexual intercourse	1.50	(0.620-3.530)	0.781	0.377
**Perception of risk of contracting HIV**				
How at risk of contracting HIV are you	1.30	(0.548-2.946)	0.310	0.577
**Age**	0.15	(0.008-2.702)	1.671	0.196
**Marital status**	2.50	(0.382-17.057)	0.937	0.333
**Religious affiliation**	1.58	(0.200-12.540)	0.190	0.663

## Discussion

Many factors contribute to the rapid spread of HIV in Cameroon including low condom use, the low socioeconomic status of women and girls, the high prevalence of other STIs, harmful socio-cultural practices and increased movement of people across borders and between urban and rural areas. At-risk populations include long-distance truck drivers and migrant populations such as road construction workers [6]. The Health Belief Model (HBM) theorizes that people's beliefs about whether or not they are at risk for a disease or health problem (HIV/AIDS) and their perceptions of the benefits of taking action to avoid it (consistent condom use), influence their readiness to take action (use condoms consistently during sex to prevent HIV/AIDS) [9, 10]. This study used the HBM as the framework to investigate the psychosocial predictors of consistent condom use among migrant road construction workers in the South-west region of Cameroon. In this study, the perception of risk of contracting HIV was low, 42.9%. Therefore, the majority of the road construction workers, 57.1% did not perceive themselves to be at risk of contracting HIV/AIDS. This majority may therefore not see the need for consistent condom use during sexual intercourse to prevent HIV transmission. The reason for the low-risk perception could be that the workers see their sexual partners as safe since they are resident in villages around the road construction sites with limitation in their movements and might therefore not be exposed to urban lifestyles and risky practices that may expose them to the risk of contracting HIV/AIDS. According to the HBM, for sexually active road construction workers to use condoms consistently, they must perceive themselves to be susceptible to the disease. From the many interventions and sensitisation programmes that have been going on since the advent of HIV, one would expectant that almost everyone should see themselves susceptible to HIV. Since Cameroon is experiencing a generalised epidemic with a prevalence of 4.3%, one would expect that the perceived susceptibility of road construction workers to HIV should be near 100%. However, the over 20% of the construction workers who did not perceive themselves to be susceptible to HIV, might not be motivated to use condom consistently during sexual intercourse to prevent the disease because they see themselves as not being at risk of contracting HIV. According to the assumptions of the HBM, for sexually active road construction workers to use condom consistently during sexual intercourse to prevent HIV transmission, they must realise that contracting HIV would have serious physical and social implications. It is when they realise the magnitude of the negative consequences of HIV/AIDS, that they would use condom consistently during sexual intercourse to prevent HIV/AIDS and avoid these negative consequences [14]. In this study, the 35.8% of the workers who did not perceive that HIV/AIDS is a deadly disease might not see the need of using condoms consistently during sex to prevent HIV transmission. This finding emphasises the necessity of strategies to increase the perception of risk of contracting HIV/AIDS among road construction workers. According to the HBM, sexually active road construction workers who perceive condom as effective in preventing HIV transmission would use it consistently during sexual intercourse. Despite the universal promotion of condom as beneficial in preventing HIV transmission, up to 37.4% of the respondents in this study did not believe in the effectiveness of condom use during sexual intercourse to prevent HIV/AIDS infection. This perception could deter these road construction workers from using condoms consistently during sexual intercourse to prevent the transmission of HIV/AIDS. Any obstacle in the use of condom could interfere with their consistent use as a means of preventing HIV/AIDS [15]. Perceived barriers refer to one's belief in the tangible and psychological costs of the advised behaviours against a condition or problem [16, 17].

According to the HBM, for workers to use condoms consistently to prevent HIV transmission they must perceive few barriers to condom use and be able to overcome these barriers. The majority of the respondents in this study, 63.8% perceived that condom decreases sexual pleasure. Therefore because of this barrier, these respondents may not use condoms consistently to prevent HIV transmission. This finding calls for concerted efforts and interventions among road construction workers to enable them to understand that the benefit of condom in preventing HIV/AIDS, outweighs any barrier they might experience in using condom since the consequences of contracting HIV are serious. Going by the tenets of the HBM, It is only when a worker realises that he has the capacity to deal with any barrier to condom use, that he would be able to use it consistently during sexual intercourse. Perceived self-efficacy for condom use is the strength of a worker's belief in his own ability to refuse sex with his partners if they refuse to use condom [17]. A worker should feel that he is capable of using condom correctly because it is that confidence that would motivate him to initiate condom use and use it consistently. In this study, up to 46.1% of the road construction workers did not have the confidence in their ability to refuse sex with their partners if they refuse to use condom. This calls for programmes and interventions to increase condom negotiation skills among migrant road construction workers. According to the HBM, migrant road construction workers who perceived themselves to be at risk of contracting HIV/AIDS, need to have confidence in their ability to use condom before they could use it consistently during sexual intercourse to prevent HIV/AIDS. Migrant workers with low condom use self-efficacy might not use condoms consistently during sexual intercourse to prevent HIV/AIDS. Perceived self-efficacy in this study was low and this was reflected in this study where only 43.5% of the sexually active migrant workers reported consistent condom usage during sexual intercourse. The finding of low condom use in this study is in agreement with other reports among migrant workers in Ethiopia where consistent condom use was reported as 46.0% [18]. This shows that sexually active migrant road construction workers are at high risk of contracting HIV/AIDS. The low percentage of consistent condom use in this study suggests that perceived barriers to using condoms may overshadow the perceived benefits of condom use and perceived self-efficacy for condom use. Therefore strategies to increase the perception of risk of contracting HIV/AIDS and to overcome barriers to condom use should be implemented in HIV/AIDS prevention programmes for migrant road construction workers in Cameroon. These programmes should also aim at increasing migrant workers' self-efficacy that they can use condoms consistently and address strategies on how to overcome barriers in negotiating condom use. Other important findings in this study were the young age of the migrant road construction workers and their low level of education. The majority of them 49.2% were aged 10-39 years, which coincides with the age group hardest hit by HIV/AIDS [19]. Also, the majority of them, 68.1%, had up to only primary level education. Their young age, coupled with their low level of education makes them more vulnerable to HIV transmission because most of them may lack sufficient knowledge regarding transmission and prevention of HIV, and also skills to negotiate condom use with their sexual partners. Since most of the migrant road construction workers are paid on the daily or weekly basis, they always have money at hand to buy sex regularly, which might put them at risk of contracting HIV because most of them do not use condom consistently. There is, therefore, the need to organize HIV/AIDS awareness programmes on a regular basis at the construction sites so that workers can be imparted with knowledge and skills to protect themselves against HIV transmission.

In this study, none of the constructs of the HBM was a significant predictor of consistent condom use. These findings are contrary to other findings among migrant workers in Benin [20] where perceived barrier was a significant predictor of consistent condom use; in Myanmar [21] and Thailand [22], where perceived susceptibility was found to be a significant predictor of consistent condom use; and in Ethiopia [20], South Africa [23] and Tanzania [24] where perceived self-efficacy was found to be significant predictor of consistent condom use. However, the results of this study are consistent with those from Myanmar [21] where perceived benefit of condom use and perceived severity were not found to be significant predictors of consistent condom use among migrant workers. The lack of significance of the constructs of the HBM as predictors of consistent condom use might be due to the low perception of risk of contracting HIV/AIDS among the road construction workers, 42.9%. This is in accordance with the assumptions of the HBM, which states that without migrant construction workers' perceptions of HIV/AIDS being a threat, there could be no resultant preventative actions against HIV/AIDS (consistent condom use). Therefore, the perceived risk of contracting HIV/AIDS is assumed to be the immediate antecedent of the consistent condom use during sexual intercourse to prevent HIV/AIDS [9, 10]. The higher migrant road construction workers' perceived risk of contracting HIV/AIDS, the higher their chances of using condoms consistently. This low perception of risk of contracting HIV among the migrant workers might have resulted in the low use of condom consistently during sexual intercourse. Therefore the hypothesis that migrant road construction workers would use condom consistently to prevent HIV transmission if they perceive a high risk of contracting HIV, was accepted at the level 0.05. Although none of the constructs of the HBM and the socio-demographic variables was a significant predictor of consistent condom use among migrant road construction workers, however, perceived susceptibility was associated with an increased likelihood of using condom consistently; perceived benefit of condom use was associated with an increased likelihood of using it consistently; perceived self-efficacy for condom use was associated with an increased likelihood of using it consistently; perceived barrier to condom use was associated with a reduced likelihood of using it consistently; perception of being at risk of contracting HIV was associated with an increased risk of using condom consistently; being a Muslim was associated with an increased likelihood of using condom consistently and being single was associated with an increased likelihood of using condom consistently (Table 3). These results call for programmes and interventions aimed at increasing the perception of risk of contracting HIV, the perceived susceptibility to HIV/AIDS, perceived benefits of condom use and perceived condom use self-efficacy among migrant road construction workers in the South-west region of Cameroon. This might result in an increase in consistent condom use.

**Limitations**: This is among the first evidence-based studies on migrant road construction workers in Cameroon that addresses the psychosocial predictors of consistent condom use. The results of the study should be interpreted with the following limitations. Firstly, the study used a cross-sectional design and thus cause-effect relationships could not be ascertained. Secondly, the study relied on self-reported information. Therefore, reports on condom use may be subject to social desirability bias. Thirdly, the study applied venue-based sampling, which may lead to selection bias. Fourthly, the sample size was small, and as such the results might not be generalised to migrant road construction workers in other parts of Cameroon. Despite these limitations, this study provides insight into the psychosocial predictors of consistent condom use among migrant road construction workers in Cameroon.

## Conclusion

Despite the limitations, this study has shown that migrant road construction workers in Cameroon are at high risk of contracting HIV/AIDS as a result of inconsistent condom use. These findings suggest that perceived barriers to using condoms may overshadow the perceived benefits of condom use and perceived self-efficacy for condom use. Therefore, strategies to increase the perception of risk of contracting HIV/AIDS, and to overcome barriers to condom use should be implemented in HIV/AIDS prevention programmes for migrant road construction workers in Cameroon. The programmes should also aim at increasing workers' self-efficacy that they can use condoms consistently and address strategies on how to overcome barriers in negotiating condom use.

### What is known about this topic

- Migration, mobility and HIV/AIDS are well-documented interlinked phenomena; high rates of HIV infections are generally found along transport routes and in regions experiencing high seasonal and long-term population mobility as that found in road construction sites;
- High mobility, isolation and working in confined environments with limited contacts, being very young adults or in sexually active age group, access to and ready availability of sex workers and inadequate access to health services, make migrant road construction works highly susceptible to HIV transmission;
- The main mode of HIV transmission in Cameroon is sexual intercourse, accounting for 90% of new infections. However, prevention is the first line of defense against HIV/AIDS and the correct and consistent use of condom is a mainstay of HIV prevention approaches.

### What this study adds

- This study adds new knowledge to the existing body of literature because it is the first study to investigate the psychosocial predictors of consistent condom use among migrant road construction workers in Cameroon;
- This study reports that consistent condom use and perception of risk of contracting HIV among migrant road construction workers in the South-west region of Cameroon are low;
- However, using the HBM as a framework, there are no significant associations between the psychosocial constructs and consistent condom use at the level 0.05; these findings could be used by policy makers and stakeholders to design and implement programmes and strategies to increase condom use among migrant road construction workers in the South-west region of Cameroon.

## Competing interests

The authors declare no competing interest.
